# Reviewing Probiotics’ effects on Major depressive disorders in older adults: a systematic analysis of randomized controlled trails

**DOI:** 10.1192/j.eurpsy.2026.11444

**Published:** 2026-07-31

**Authors:** M. Arts, M. Zeilstra, K. M. Zeilstra-Kalt, L. D. Jonge

**Affiliations:** 1 Geriatric Psychiatry and Neuropsychiatry; 2 Lifestyle Medicine, GGZ WNB, Halsteren; 3 Integrative Medicine, Center de Wetering, Leeuwarden; 4 Integrative Medicine, Leonardo Scientific Research Institute, Groningen, Netherlands

## Abstract

**Introduction:**

Major depressive disorder (MDD) is a prevalent mental health condition impacting millions globally. It is characterized by persistent low mood, loss of interest, and associated symptoms. Pharmacological treatments typically target neurotransmitter activity but often show delayed efficacy and side effects. Increasing evidence highlights the bidirectional “brain-gut axis” as a pathway linking microbiota alterations to psychiatric disorders. Preclinical studies suggest probiotics may influence mood via modulation of neurotransmitter activity. While causality remains unclear, probiotics represent a promising adjunctive strategy for MDD. This systematic review evaluates current randomized controlled trials (RCTs) on probiotics for older adults with MDD, aiming to clarify their therapeutic utility.

**Objectives:**

To systematically assess the efficacy of probiotics in reducing depressive symptoms among older adults with MDD, and to evaluate their potential role as an adjunctive therapy in clinical practice.

**Methods:**

A systematic literature search was conducted in PubMed, Embase, and PsycINFO up to April 2024. Eligible studies were randomized controlled trials investigating the effect of probiotics in adults aged 60 years and older with a diagnosis of moderate-to-severe MDD according to DSM-IV or DSM-V criteria.

**Results:**

Six RCTs, comprising a total of 389 participants, met inclusion criteria. Sample sizes ranged from 40 to 102, with treatment durations between 8 and 12 weeks. Various probiotic strains were investigated, most frequently Lactobacillus and Bifidobacterium species, administered either as monotherapy or adjunctive to antidepressants. Across all included studies, participants receiving probiotics demonstrated greater improvements in depressive symptom scores compared to placebo groups. However, only three trials reported statistically significant reductions in depression severity after probiotic administration. Heterogeneity in study design, strain composition, dosage, and outcome measures limited comparability. Notably, adverse effects were minimal and no serious safety concerns were reported, supporting the tolerability of probiotic treatment.

**Conclusions:**

Evidence from RCTs suggests probiotics may reduce depressive symptoms in older adults with MDD, though findings remain inconsistent. Given their favorable safety profile and tolerability, probiotics represent a promising adjunctive therapy. Further large-scale, well-designed trials are required to confirm efficacy, clarify mechanisms, and explore interactions with standard antidepressant treatments.

**Disclosure of Interest:**

None Declared

